# New experimental setup for the measurement of cleaning efficacy and force of interdental aids in 3D-reproduced interdental areas

**DOI:** 10.1186/s12903-020-01129-z

**Published:** 2020-05-08

**Authors:** Christian Graetz, Johanna Rabe, Kristina Schoepke, Susanne Schorr, Antje Geiken, David Christofzik, Thomas Rinder, Christof E. Dörfer, Sonja Sälzer

**Affiliations:** 1 Clinic of Conservative Dentistry and Periodontology, University of Kiel, Arnold-Heller-Str. 3, Haus B, 24105 Kiel, Germany; 2 Institute of Mechatronics, Computer Science and Electrical Engineering, Kiel University of Applied Sciences, Kiel, Germany

**Keywords:** In vitro procedure, Mechanical plaque control, Rubber bristle interdental cleaner, Cleaning efficacy, Resistance to insertion, 3D printing

## Abstract

**Background:**

Interdental rubber picks (IRP) have become a frequent and convenient alternative for interdental cleaning. However, only little evidence exists supporting the effectiveness of newer designs available on the market. Therefore, a new in vitro model was evaluated to measure the experimental cleaning efficacy (ECE), as well as the force needed for insertion and during the use of IRP, with high reproducibility.

**Methods:**

Five different sizes of commercially marketed IRP with elastomeric fingers (IRP-F) (GUM SOFT-PICKS® Advanced, Sunstar Deutschland GmbH, Schönau, Germany) or slats (IRP-S) (TePe EasyPick™, TePe D-A-CH GmbH, Hamburg, Germany) were tested. Interdental tooth surfaces were reproduced by a 3D-printer (Form 2, Formlabs Sommerville, MA, USA) according to human teeth and matched to morphologically equivalent pairs (isosceles triangle, concave, convex) fitting to different gap sizes (1.0 mm, 1.1 mm, 1.3 mm). The pre−/post brushing situations at interdental areas (standardized cleaning, computer aided ten cycles) were photographically recorded and quantified by digital image subtraction to calculate ECE [%]. Forces were registered with a load cell [N].

**Results:**

IRP-F have to be inserted with significant higher forces of 3.2 ± 1.8 N compared to IRP-S (2.0 ± 1.6 N; *p* < 0.001) independent of the size and type of artificial interdental area. During cleaning process IRP-S showed significantly lower values for pushing/pulling (1.0 ± 0.8 N/0.5 ± 0.4 N) compared to IRP-F (1.6 ± 0.8 N/0.7 ± 0.3 N; *p* < 0.001) concomitant to significantly lower ECE (19.1 ± 9.8 vs. 21.7 ± 10.0%, *p* = 0.002). Highest ECE was measured with largest size of IRP-F/IRP-S independent the morphology of interdental area.

**Conclusions:**

New interdental cleaning aids can be tested by the new experimental setup supported by 3D printing technology. Within the limitations of an in vitro study, IRP-F cleaned more effectively at higher forces compared to IRP-S.

## Background

Beside a lot of efforts developing better toothbrushes, up to date their bristles do not reach the interproximal surfaces of teeth efficiently [1]. This seems important as interdental sites present the highest risk of plaque accumulation and the highest prevalence of caries and infrabony pockets in an adult population [2]. Therefore, additional devices are necessary to penetrate between adjacent teeth [3]. Moreover, current systematic reviews indicate that additionally to tooth brushing, cleaning with different interdental brushes versus flossing is superior for prevention and treatment of gingivitis than tooth brushing only [4, 5]. Although, interdental cleaning with wood sticks can significantly reduce bleeding on probing and gingivitis, they do not reduce plaque parameters [5, 6]. More controversially discussed are the recently developed interdental rubber picks, which seem to be able to reduce plaque but data for gingivitis are inconsistent [7, 8]. Hence it’s not surprising that a currently published Cochrane review did not find any difference by very low-certainty evidence for interdental brushes or flossing versus interdental rubber picks [6]. According to the knowledge of the authors so far, no studies have been published comparing clinical efficacy of differently designed rubber picks. The authors of this review concluded [6] that the available evidence for interdental rubber picks is limited and inconsistent.

In addition, the absolute validity and reliability in vivo regarding the interdental cleaning efficacy is hampered by the accuracy with which the tested parameters may be determined [9]; e.g. residual interdental plaque is not directly measurable resulting in a lack of precision and consistency of the data. On the other side, the majority of in vitro studies used standardized isosceles triangle interdental space, keeping the force used to penetrate the interdental space standardized or to measure experimental cleaning efficacy (ECE) in a reproduceable manner. However, by doing so, they neglect more clinically relevant morphologies, such as convex or concave shapes of the proximal root surfaces [10].

Hence, the primary aim of the present study was to develop a new experimental setup in order to test in vitro*,* under standardised, controlled and reproducible conditions, the interdental ECE and the cleaning force. A further aim was to compare two different types of rubber picks - elastomeric fingers IRP-F and elastomeric slats IRP-S with regard to interdental ECE and the cleaning force.

## Methods

### Experimental setup

**Fig. 1:**
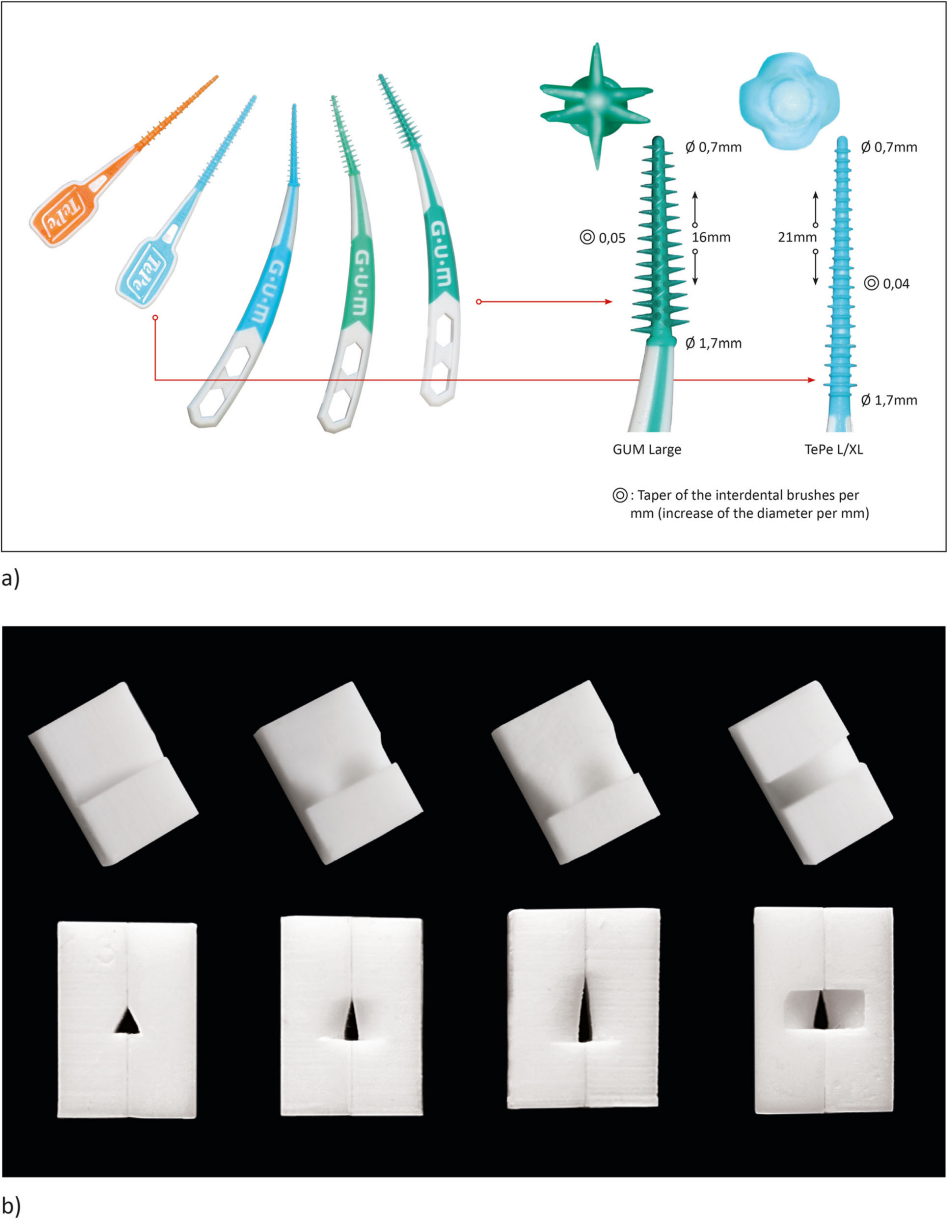
**a** Illustration of the test devices for interdental cleaning (from left): interdental rubber picks with slat-design (IRP-S) and with finger-design (IRP-F) (magnification showed in detail the different design of interdental rubber picks, each the largest test diameter). IRP-S size S/M ISO 1-4, IRP-S size L/XL ISO 3-6, IRP-F size small ISO1, IRP-F size regular ISO 2 and IRP-F size large ISO 4. The working part of the IRP-S is 21 mm with a taper of 0.04 of the core, while the IRP-F is 16 mm with a taper of 0.05 of the core. **b** Illustration of four different morphologies of artificial interdental areas (from left: isosceles triangle, concave space of 3 mm height, concave space of 5 mm height and convex; all shown morphologic in size 1.3 mm)

In this in vitro study, two different designs of interdental rubber picks were tested in all available sizes (Fig. 1a); the IRP-F with elastomeric fingers in small (ISO 1), regular (ISO 2) and large size (ISO 4) (GUM SOFT-PICKS® Advanced, Sunstar Deutschland GmbH, Schönau, Germany) and the other one IRP-S with elastomeric slats in extra-small/small (ISO 1-4) and medium-large/large (ISO 3-6) (TePe D-A-CH GmbH, Hamburg, Germany). The major difference between the two IRPs is the shape of the bristles, with more pointed bristles (F = fingers) for IRP-F and flatter bristles (S = slats) for IRP-S.

**Fig. 2:**
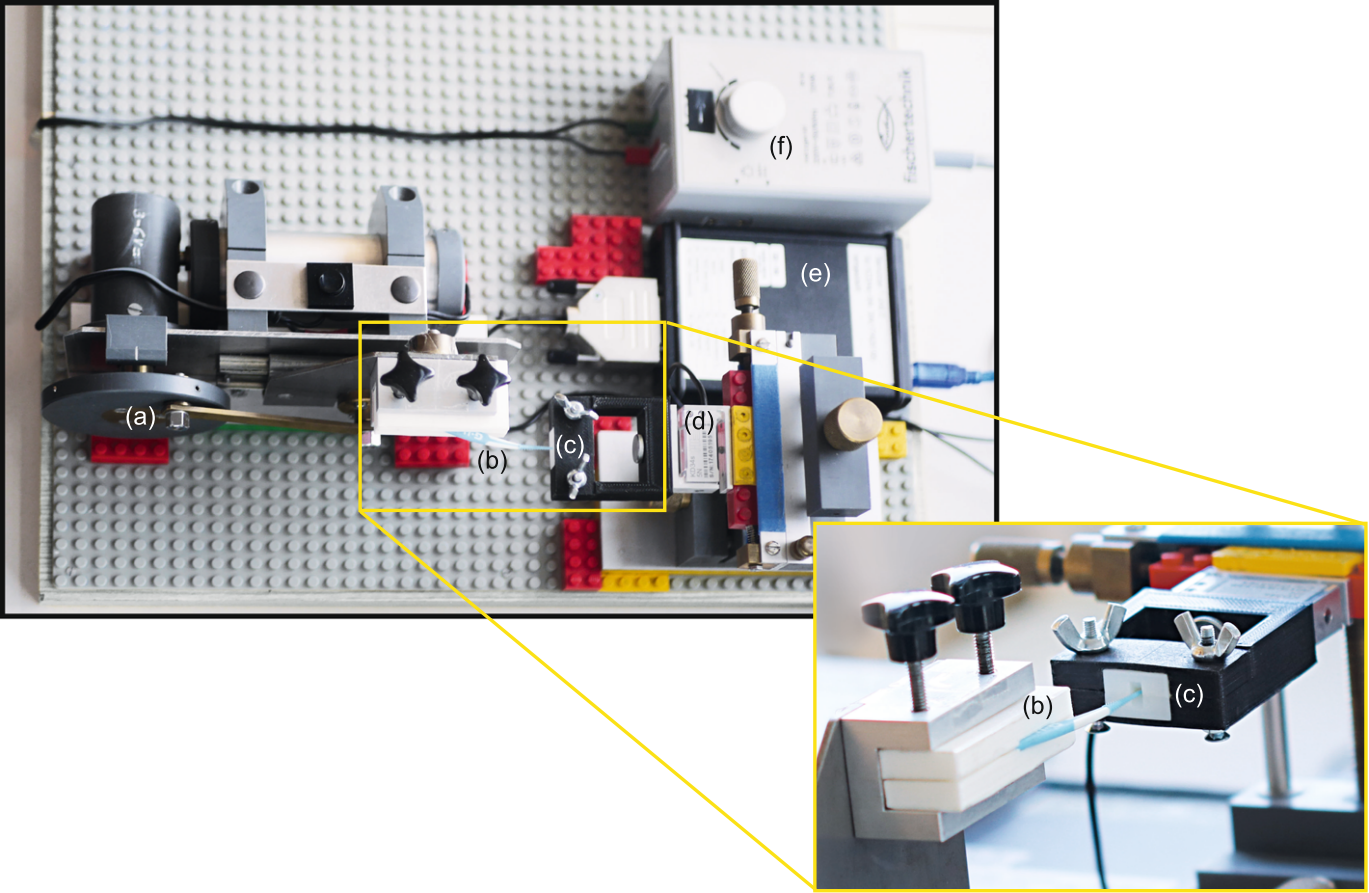
Overview of experimental setup mechanical device, which converts rotation into a linear motion (**a**) moves the (**b**) test products into the (**c**) artificial interdental area (details shown in the separate magnification). The (**d**) digital load cell records the applied force longitudinally and documents it in a table chronological (not shown), (**e**) control unit for motion and load cell and (**f**) the electric transformer

With the help of a computer software (Autodesk Fusion 360, Autodesk Direct Limited, Hampshire, United Kingdom) and in vivo data of interdental morphologies [10–12], 3D composite replicas were designed and printed with a layer thickness of 25 μm, resulting in a corresponding surface roughness. Using a stereolithography (SLA) 3D printer (Form 2, Formlabs Sommerville, MA, USA) with a laser to cure a liquid photopolymer resin (White Resin V04 (RS-F2-GPWH-04), Formlabs, Sommerville, MA, USA), it was possible to print reproducible geometries with a high degree of accuracy [13]. The SLA printing method used, resulted in different resolutions between the XY-axes and the Z- axis. In the Z-axis, a maximum roughness of 25 μm was achieved. Depending on the geometry of the object, the roughness in the XY-axis varied between 8 and 25 μm. These settings were considered when aligning the objects for printing, in order to achieve the highest possible accuracy. The replicas were fixed pairwise in a socket with an embedded load cell (KD34s, ME-Meßsysteme GmbH Hennigsdorf, Germany; measuring range: ±500mN with precision class of 0.1%). This allowed a continuous measuring of the applied forces during ten cleaning cycles and an automatic documentation in a table (Microsoft Excel 2016, Microsoft Corporation, Redmond, WA, USA), as well as the removal and replacement of the adjacent teeth surfaces in a reproducible manner (Fig. 2). Due to the background noise of the load cell between two cleaning cycles, only data > 0.09 N were included.

**Fig. 3:**
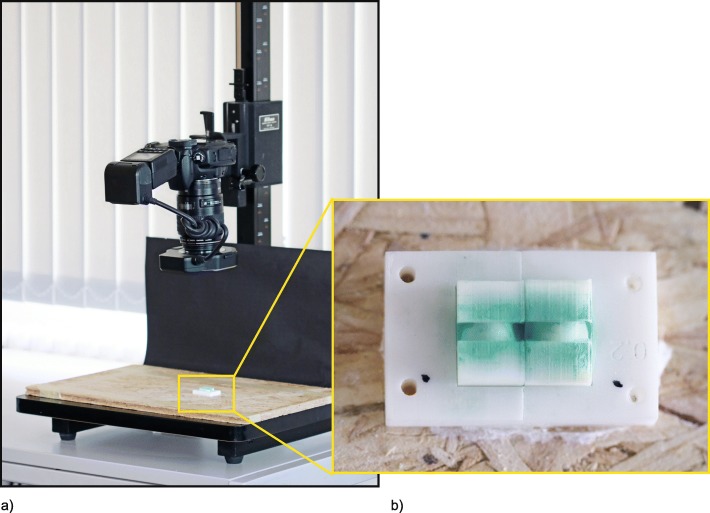
(**a**) Setup for photographic documentation and (**b**) detail of one convex artificial interdental area (flipped, central is the previously site of insertion of the test devices)

**Fig. 4:**
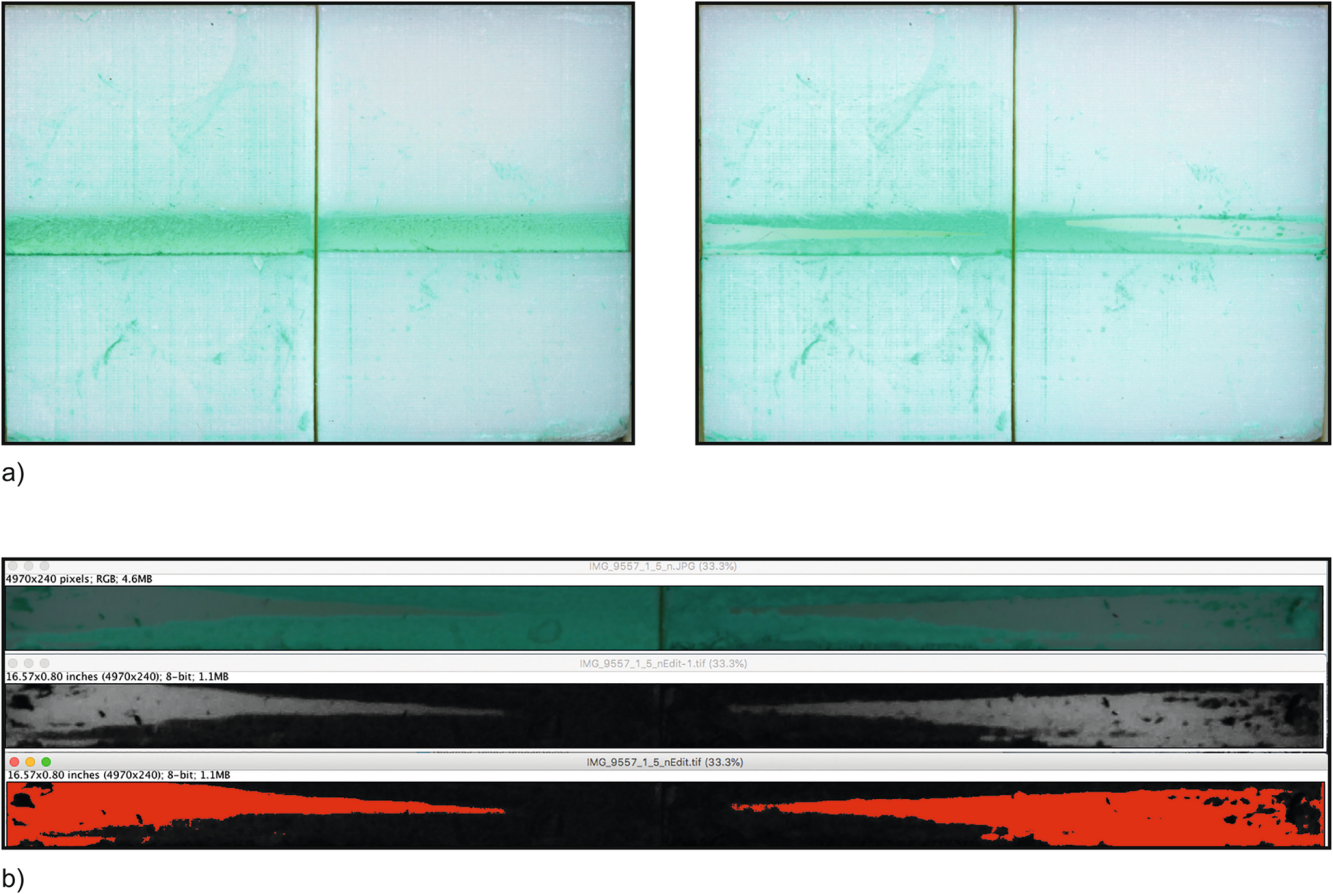
**a**) A sample of artificial interdental area (isosceles triangle) to illustrate the standardized photographic documentation before and after cleaning by the test products. The same test blocks with the decolorized, purified areas of the simulated interdental area are again photographed in order to subsequently perform an (**b**) evaluation of the cleaned surfaces in percent by a digital image subtraction (Image J, NIH, Bethesda, USA) to calculate the experimental cleaning efficacy (ECE)

IRP-F are recommended by the manufacturer for interdental areas of size 0.8–1.5 mm (IRP-S: 0.7–2.0 mm). Therefore, three interdental gap sizes of 1.0 mm (small), 1.1 mm (medium) and 1.3 mm (large) were created to test the different sizes of IRP. Furthermore these 3 sizes were created in four morphologies (isosceles triangle, convex, concave space of 3 mm height and concave space of 5 mm height), resulting in 12 different artificial interdental areas (Fig. 1b). The different sizes of the interdental area were created in relation to the three sizes of the test device IRP-F in small, regular and large and adjusted to the two sizes of the IRP-S in extra-small/small and medium-large/large. Subsequently, the interdental area replicas were stained by one investigator (J.R.) with Occlu Spray Plus (Hager & Werke, Duisburg, Germany) as described in previous studies [14, 15]. A standardized powder thickness (mean ± SD: 20 ± 5 μm; supporting information in figure S1) was ensured by a standardized procedure and appropriate time protocol. The baseline surface was digitally photographed (Canon EOS 400D Digital, Uxbridge, United Kingdom) and documented. Afterwards, a mechanical device, which converts rotation into a horizontal motion, moved the interdental cleaning aids with a controlled speed ten times (10x for- and backward) into the artificial interdental area (Fig. 2). Therefore, all different interdental cleaning aids could be tested in a reproducible manner, since each cleaning aid was inserted into the same point and was moved in the same direction. After the test, all artificial interdental area replicas were again photographed (Fig. 3) in order to subsequently perform an evaluation of ECE by digital image subtraction (Image J, NIH, Bethesda, USA) (Fig. 4).

The experimental cleaning efficacy ECE was determined as the difference of simulated biofilm before and after cleaning the interdental area in %. The measured force was divided into two intervals: First, the force necessary to insert the test products into the artificial interdental area (insertion force IF) and second, the force necessary for cleaning (pushing and pulling).

### Statistical analysis

A power calculation for the determination of the sample size was based on the results of a previously published in vitro study on the cleaning efficacy (percent of removed simulated biofilm) and resistance to insertion of two different interdental brushes [15]. According to this sample size calculation (sub-group analysis was considered beforehand), we found *n* = 25 samples per group as sufficient to detect 5 % difference for experimental cleaning efficacy between the groups of different test products with a power of 80%.

Means, percentages and standard deviations were calculated using Microsoft Excel (Microsoft Excel 2016, Microsoft Corporation, Redmond, WA, USA). Values were mostly ordinal (yes/no) or metric and with the use of Microsoft Excel, crosstabs have been drawn up. For statistical analysis, data were entered in SPSS Statistics (SPSS Statistics 24, IBM, Chicago, IL, USA). Normal distribution of the recorded values was tested with the Kolmogorov–Smirnov/Lilliefors test. Mean values of the ECE and of the forces, separated for IF, push and pull, were calculated for every tested product and type/gap size of artificial interdental area separately. The differences between products or artificial interdental areas were tested for statistical significance by ANOVA and the paired t-test. Statistical significance was assumed if *p* ≤ 0.05. All types of IRPs, with their different sizes and morphologies, were compared to each other.

## Results

Results for ECE and force measurements are summarized for both groups of different interdental rubber picks in Table 1. In 79 out of 675 tests performed, the analysis of the ECE was not possible and the data could not be used in the final assessment (*n* = 596). In only three out of 675 tests performed, the data for force measurements were missing and a total of 672 data sets could be analyzed.

**Table 1:** Overall results of experimental cleaning efficacy (ECE in %) and forces (in N)

	**Type of interdental area**					
	**isosceles triangle**	**convex**	**concave**	**isosceles triangle vs. convex**	**isosceles triangle vs. concave**	**convex vs. concave**
**ECE in %**	31.14 ± 7.82	14.76 ± 7.55	17.81 ± 7.67	*p* < 0.001	*p* < 0.001	*p* < 0.001
**mean IF in N**	2.47 ± 1.30	1.80 ± 1.21	2.88 ± 2.06	*p* = 0.000	*p* = 0.023	*p* < 0.001
**mean push in N**	1.20 ± 0.57	1.04 ± 0.65	1.42 ± 0.98	*p* = 0.020	*p* = 0.010	*p* < 0.001
**mean pull in N**	0.59 ± 0.27	0.55 ± 0.29	0.64 ± 0.39	*p* = 0.201	*p* = 0.163	*p* = 0.008
	**Size of interdental area**					
	**1.0 mm**	**1.1 mm**	**1.3 mm**	**1.0 mm vs. 1.1 mm**	**1.0 mm vs. 1.3 mm**	**1.1 mm vs. 1.3 mm**
**ECE in %**	18.74 ± 12.49	18.38 ± 7.54	24.53 ± 8.59	*p* = 0.719	*p* < 0.001	*p* < 0.001
**mean IF in N**	2.47 ± 1.62	2.15 ± 1.66	3.05 ± 1.95	*p* = 0.038	*p* = 0.001	*p* < 0.001
**mean push in N**	1.22 ± 0.72	1.10 ± 0.82	1.55 ± 0.90	*p* = 0.115	p < 0.001	*p* < 0.001
**mean pull in N**	0.59 ± 0.31	0.56 ± 0.39	0.67 ± 0.28	*p* = 0.385	*p* = 0.011	*p* = 0.001
	**Size of the interdental rubber picks**					
	**small**	**regular**	**large**	**small vs. regular**	**small vs. large**	**regular vs. large**
**ECE in %**	17.90 ± 11.0	17.68 ± 7.46	23.96 ± 8.33	*p* = 0.861	*p* < 0.001	*p* < 0.001
**mean IF in N**	1.89 ± 1.58	2.22 ± 1.04	3.29 ± 1.90	*p* = 0.049	*p* < 0.001	*p* < 0.001
**mean push in N**	0.95 ± 0.71	1.05 ± 0.45	1.70 ± 0.90	*p* = 0.183	*p* < 0.001	*p* < 0.001
**mean pull in N**	0.47 ± 0.31	0.56 ± 0.21	0.77 ± 0.34	*p* = 0.007	*p* < 0.001	*p* < 0.001

Force for insertion into the artificial interdental area as well as during ten cleaning cycles (mean push/pull) according the three different morphologies (isosceles triangle, convex, concave) and sizes (1.0 mm, 1.1 mm, 1.3 mm) of artificial interdental area. We assumed *p* < 0.05 to be statistically significant (ANOVA, paired t-test, two sided)

### Cleaning efficacy

In general, mean ± SD ECE for all tested interdental rubber picks was 20.3 ± 9.9% (range: 5.2–64.2%) independent of size and type of artificial interdental area. When stratifying, we found the highest ECE for the isosceles triangle interdental morphology (31.1 ± 7.8%; *p* < 0.001), the biggest gap size of artificial interdental area (1.3 mm; 24.5 ± 8.6%; p < 0.001), as well as for the largest tested interdental rubber picks (Large; 24.0 ± 8.3%; p < 0.001), respectively (Table 1).

Comparing the two test products, IRP-F showed with 21.7 ± 10.0% significantly better results for ECE as IRP-S (19.1 ± 9.8%; *p* = 0.002). After stratification for type of the artificial interdental area (isosceles triangle vs. convex vs. concave), only significant differences between IRP-F and IRP-S for ECE of the convex interdental area (16.7 ± 9.6% vs. 13.1 ± 4.8%; *p* = 0.003) and the concave interdental area (19.1 ± 7.7% vs. 16.9 ± 7.6%; *p* = 0.013) were detectable, but not for the isosceles triangle interdental area (31.2 ± 7.2% vs. 31.1 ± 1.6%; *p* = 0.944) (Table 2). For large gap sizes of artificial interdental area, a significant difference between both types of interdental rubber picks was detectable, favoring IRP-F (28.2 ± 6.8 vs. 20.5 ± 8.6; *p* < 0.001) (Table 2).

**Table 2:** Subgroup results (mean ± SD) of experimental cleaning efficacy (ECE in %) and forces (in N)

**Morphology and size of interdental area**	**Height of concave interdental area**	**ECE in %**		***p*-value**	**insertion force in N**		***p*-value**	**mean push in N**		***p*-value**	**mean pull in N**		***p*-value**
		IRP-F	IRP-S		IRP-F	IRP-S		IRP-F	IRP-S		IRP-F	IRP-S	
**Isosceles triangle**		31.18 ± 7.22	31.09 ± 1.55	p = 0.944	3.34 ± 1.09	1.60 ± 0.83	p < 0.001	1.55 ± 0.50	0.85 ± 0.38	p < 0.001	0.73 ± 0.26	0.45 ± 0.19	p < 0.001
**convex**		16.69 ± 9.61	13.08 ± 4.58	p = 0.003	2.30 ± 1.12	1.47 ± 1.18	p < 0.001	1.25 ± 0.6	0.87 ± 0.64	p < 0.001	0.65 ± 1.73	0.47 ± 0.33	p < 0.001
**concave**	3 mm	21.08 ± 6.59	19.54 ± 5.75	*p* = 0.131	5.20 ± 1.84	3.39 ± 1.95	p < 0.001	2.45 ± 0.90	1.67 ± 0.97	p < 0.001	0.96 ± 0.23	0.8 ± 0.45	*p* = 0.004
	5 mm	17.32 ± 8.17	13.58 ± 8.20	p = 0.007	1.98 ± 0.88	1.33 ± 0.87	p < 0.001	1.01 ± 0.46	0.71 ± 0.43	p < 0.001	0.48 ± 0.21	0.35 ± 0.21	p < 0.001
	3 + 5 mm	19.10 ± 7.67	16.86 ± 7.55	p = 0.013	3.60 ± 2.16	2.36 ± 1.83	p < 0.001	1.73 ± 1.02	1.19 ± 0.89	p < 0.001	0.72 ± 0.33	0.57 ± 0.42	p < 0.001
**1.0 mm**		19.14 ± 11.7	18.36 ± 13.22	*p* = 0.213	3.23 ± 1.73	1.73 ± 1.10	p < 0.001	1.56 ± 0.77	0.88 ± 0.47	p < 0.001	0.74 ± 0.32	0.44 ± 0.21	p < 0.001
**1.1 mm**		17.68 ± 7.46	18.82 ± 7.58	*p* = 0.844	2.22 ± 1.04	2.11 ± 1.93	p = 0.605	1.05 ± 0.44	1.13 ± 0.98	p < 0.001	0.56 ± 0.21	0.57 ± 0.47	p < 0.001
**1.3 mm**		28.17 ± 6.82	20.53 ± 8.59	p < 0.001	4.13 ± 1.96	1.97 ± 1.19	p < 0.001	2.10 ± 0.90	1.04 ± 0.54	p < 0.001	0.82 ± 0.24	0.53 ± 0.25	p < 0.001

Force for insertion (IF) into the artificial interdental area as well as during ten cleaning cycles (mean push/pull) for cleaning different types (isosceles triangle, convex, concave) and sizes (1.0 mm, 1.1 mm, 1.3 mm) of the interdental area separated for the tested interdental rubber picks with fingers-design (IRP-F) versus slats-design (IRP-S). We assumed p < 0.05 to be statistically significant (ANOVA, paired t-test, two sided)

### Insertion forces and forces for cleaning

Table 2 provides an overview of the necessary forces for cleaning for both types of interdental rubber picks. The overall mean pushing force was assessed as 1.3 ± 0.8 N (mean ± SD) and pulling force as 0.6 ± 0.3 N, independent of the size and type of artificial interdental area. The insertion force was found higher for both types of interdental rubber picks with 2.5 ± 1.8 N, whereas IRP-S (2.0 ± 1.6 N) showed significant (*p* < 0.001) lower values than IRP-F (3.2 ± 1.8 N), independent of the gap size and type of artificial interdental area. Accordingly, the necessary insertion force for IRP-S was always lower (Table 2) with the exception of the 1.1 mm gap size of artificial interdental area (IRP-F vs. IRP-S: 2.2 ± 1.0 vs. 2.1 ± 1.9; *p* = 0.605).

During the cleaning procedure of ten cycles, the largest size of interdental rubber picks in the biggest gap size of the artificial interdental area led to the highest mean pushing and pulling force (*p* ≤ 0.011), whereas the highest necessary pushing force were found for the concave interdental area (*p* ≤ 0.010).

Overall, IRP-S showed significant lower forces (pushing/pulling) during cleaning of the artificial interdental area (1.0 ± 0.8 N / 0.5 ± 0.4 N) as IRP-F (1.6 ± 0.8 N / 0.7 ± 0.3 N; *p* < 0.001) (Table 2). After stratification for the type of the artificial interdental area (isosceles triangle vs. convex vs. concave) as well as for the gap size (1.0 mm vs. 1.1 mm vs. 1.3 mm) IRP-S showed in all tested sceneries significantly lower forces (*p* ≤ 0.001).

## Discussion

With the help of the newly developed in vitro procedure it could be demonstrated, that interdental rubber picks (IRP) with small elastomeric fingers and higher taper (IRP-F) had a significantly better cleaning efficacy as interdental rubber picks with lower elastics slats and lower taper (IRP-S). The longer fingers might be able to adapt better to the tooth surfaces compared to flatter less elastic slats. A further reason for the better results for ECE of IRP-F might be that the artificial interdental area sizes (e.g. 1.0, 1.1, 1.3 mm) were based on the available IRP-F and, hence, the IRP-S fitted a little bit less. Therefore, the size of the IRP needs to be exactly chosen to the present interdental area, as it is established for interdental brushes. Our sub-analysis of different morphologies of artificial interdental areas demonstrated a difference between the IRP designs only for convex and concave, but not for isosceles triangle shaped interdental areas. Additionally, looking at larger gap size of the artificial interdental space, the cleaning efficacy did improve for both tested designs, whereas significant better results were only found for IRP-F. As a lot of factors affect the cleaning efficacy of interdental brushes, e.g. design, material or length and diameter [16], the relative dimension of size of the interdental brushes in relation to the artificial interdental space seems to be very important [17]. Unfortunately, most studies do not mention the size of interdental brushes used [17]. From a technical point of view, the efficacy of cleaning will improve with the contact area between the interdental brushes and the tooth surface, which correlates to an increasing application force as indicted by our results and could be another explanation for the better results for IRP-F. The insertion force of round interdental brushes remains constant in a more parallel-walled interdental area, whereas in an equilateral triangular shaped interdental area, the necessary force will increase more with greater interdental brush dimensions. In contrast, the ECE decreased with smaller sized interdental brushes [11]. In analogy, the design of the tested IRP-F compared with IRP-S could be the reason for the better performance in our study, due to the higher contact area between the more elastic rubber fingers and the tooth surface as well as the higher taper. Maybe, the ECE could further enhance if the interdental rubber picks will be used in an angulated direction toward the occlusal plane and/or from both sides of the artificial interdental space. The main effort of this experimental setup was to enable the most uniform testing conditions as possible. Angulated movements are more demanding and need a greater effort (e.g. both tooth surfaces must be cleaned separately), which was reported by a lower scoring of dental floss in term of applicability compared to IRP-F [11].

In line with the better cleaning results of IRP-F, our in vitro results indicate a requirement for significantly higher pushing and pulling forces to clean the interdental area in all simulated sceneries. However, the present study did not compare the insertion forces of IRP to interdental brushes. Eventually, a thin nylon filament of a conventional interdental brushes bends with a smaller resistance than an elastomeric rubber finger of an interdental rubber pick. In addition, the rough surface of the interdental rubber picks, especially in contact with the artificial tooth surface simulated in our study (~ 25 μm versus ~ 10 μm of natural enamel [18]), will deform and create a greater resistance under usage, than the surface of the smooth nylon filaments. With up to 5 N necessary to insert the tested interdental rubber picks in a concave interdental area, interdental rubber picks do not seem to be the appropriate interdental cleaning aid for this type of interdental space. The advantage of interdental rubber picks is their wireless construction – and both tested designs, showed high primary stability without bending or fracturing of the core in our test. It can be hypothesized that with interdental rubber picks it will be easier for the patient to find the entrance in the interdental space as no discomfort in contact with the tooth surface or marginal gingiva is expected. Correspondingly, participants in all in vivo investigation’s found interdental rubber picks to be significantly more comfortable to use than interdental brushes [19–22].

Interdental rubber picks are more and more promoted and developed and could be seen as further technological evolution of interdental brushes. Instead of metal core or nylon filaments, they have small elastomeric fingers or slats protruding perpendicularly from a plastic core [19]. Whereas, by the means of a recently published meta-review [5], the highest evidence exists stating that interdental cleaning with interdental brushes is the most effective method of interdental plaque removal, only low evidence for the newer interdental rubber picks exist to date. The few published studies found no statistically significant difference between the interdental rubber picks and conventional interdental brushes or flossing, neither for gingivitis scores nor for plaque scores [19–21].

Contrary to these clinical investigations, our study focuses on the development of a standardized reproducible procedure to measure the cleaning efficacy and necessary cleaning force of interdental rubber picks and not on the clinical application of this newer interdental aid in comparison to conventional interdental brushes. Although the presented in vitro data cannot be directly applied to a clinical situation, the model has some advantages.

Currently, no method exists to quantitatively assess the interdental plaque in a clinical situation in vivo. Plaque removal of interdental aids assessed on the visible surface of the teeth might be masked by differences in the general oral hygiene procedures applied by the patients (e.g. tooth brushing routine and technique). Moreover, in an in vitro study, the size of interdental brushes can be chosen more appropriately according to the defect morphology as in a clinical study. However, an appropriate size is of major importance for the cleaning efficacy. Hence, inappropriately chosen interdental brushes might be a cause for the lack of statistical difference between various interdental cleaning aids [23]. Consequently, only in in vitro set-ups, like in the chosen one, a standardized measurement of cleaning efficacy is possible [16]. The reproduced 3D printed replicas [13] allowed with high precision the differentiation between the ECE of different surface morphologies such as plane, concave and convex surfaces [24].

However, the in vitro set-up has several limitations for extrapolating these data to a patient.

It is not known if the presented results using powder or varnish on resin models to assess cleaning efficacy and forces is comparable to real dentate plaque on enamel or cementum. Also, in the present study the IRP were moved in a straight direction into the interdental space for better reproducibility. In a patient’s mouth this ideal insertion is not always possible due to space limits and constraints. However, as long as no method to measure interdental plaque in vivo exists*,* the presented experimental set-up seems to be a valid method to measure interdental ECE and force of interdental cleaning aids. In conclusion, the advantage of this in vitro comparison is the reproducible investigation under standard conditions, which is however rarely reproducible in vivo, particularly with regard to the different anatomies and the periodontal tissues.

Moreover, further devices including different designs of interdental brushes should be tested in future studies for better comparability.

## Conclusions

New interdental cleaning aids can be tested by the new reproducible experimental setup supported by 3D technology mimicking a natural clinical situation. The method proved to be accurate and precise due to the computerized evaluation strategy. Within the limitations of this in vitro study, both types of interdental rubber picks successfully removed around 20% of the simulated biofilm in artificial interdental areas of different size and morphology. IRP-F cleaned more effectively at higher forces compared to IRP-S. Cleaning efficacy and force correlate positively, whereas both depend significantly on interdental rubber picks’ design and size. Hence, the correct choice with regard to the design and size of the IRP seems to be important. However, these in vitro data have to be verified by clinical studies.

## Supplementary information

**Additional file 1: Figure S1.** Illustration (magnification 65x) of the replicas’ surface of the interdental area (White Resin V04 (RS-F2-GPWH-04), Formlabs, Sommerville, MA, USA) and powder thickness (Occlu Spray Plus, Hager & Werke, Duisburg, Germany) for biofilm simulation (inner field magnifications in 500x and 1000x for details of the powder thickness and surface roughens).

## Data Availability

The datasets used and/or analysed during the current study are available from the corresponding author on reasonable request.

## Abbreviations

IRP: Interdental rubber picks
IRP-F: Interdental rubber picks with elastomeric fingers
IRP-S: Interdental rubber picks with slats
ECE: Experimental cleaning efficacy
