# Supplementary figures and images for: New experimental setup for the measurement of cleaning efficacy and force of interdental aids in 3D-reproduced interdental areas
Source: BMC Oral Health. 2020 May 8;20:136. doi: 10.1186/s12903-020-01129-z (PMC7206737; doi:10.1186/s12903-020-01129-z)

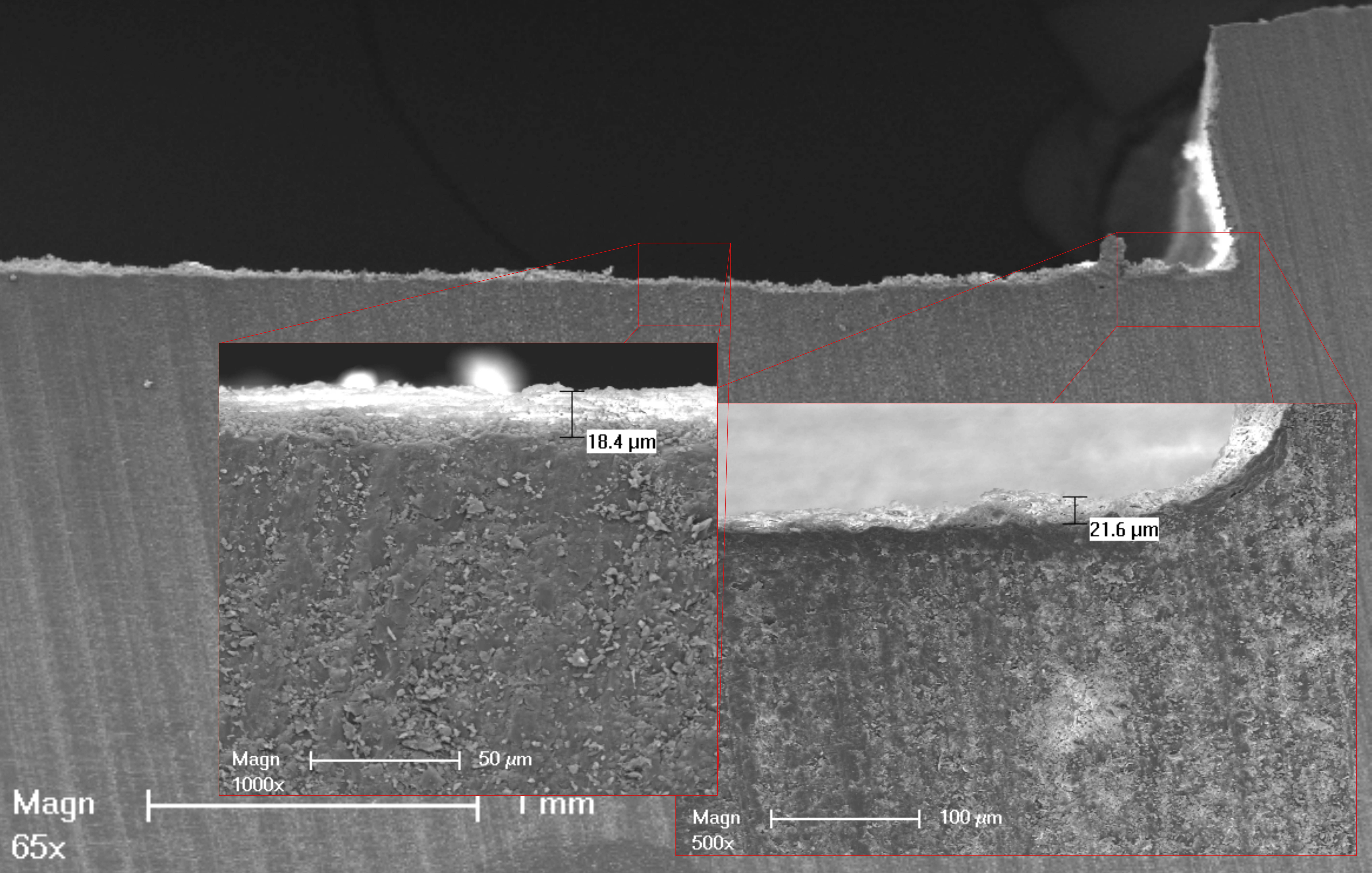

18.4  $\mu\text{m}$

Magn  
1000x

50  $\mu\text{m}$

Magn  
65x

1 mm

21.6  $\mu\text{m}$

Magn  
500x

100  $\mu\text{m}$

Supplement: Supplementary file 1 — Additional file 1: Figure S1. Illustration (magnification 65x) of the replicas’ surface of the interdental area (White Resin V04 (RS-F2-GPWH-04), Formlabs, Sommerville, MA, USA) and powder thickness (Occlu Spray Plus, Hager & Werke, Duisburg, Germany) for biofilm simulation (inner field magnifications in 500x and 1000x for details of the powder thickness and surface roughens). [file 12903_2020_1129_MOESM1_ESM.pdf]
